# Probing gigahertz coherent acoustic phonons in TiO_2_ mesoporous thin films

**DOI:** 10.1016/j.pacs.2023.100472

**Published:** 2023-03-04

**Authors:** E.R. Cardozo de Oliveira, C. Xiang, M. Esmann, N. Lopez Abdala, M.C. Fuertes, A. Bruchhausen, H. Pastoriza, B. Perrin, G.J.A.A. Soler-Illia, N.D. Lanzillotti-Kimura

**Affiliations:** a Université Paris-Saclay, CNRS, Centre de Nanosciences et de Nanotechnologies, 91120 Palaiseau, France; b Institute for Physics, Carl von Ossietzky University of Oldenburg, 26129 Oldenburg, Germany; c Instituto de Nanosistemas, Escuela de Bio y Nanotecnologías, Universidad Nacional de San Martín-CONICET, Buenos Aires, Argentina; d Gerencia Química, Inst. de Nanociencia y Nanotecnología, CNEA-CONICET, Buenos Aires, Argentina; e Centro Atómico Bariloche, Inst. de Nanociencia y Nanotecnología, CNEA-CONICET, Rio Negro, Argentina; f Sorbonne Université, CNRS, Institut des NanoSciences de Paris, INSP, F-75005 Paris, France

**Keywords:** Acoustic phonons, Mesoporous thin films, Picosecond ultrasonics, Pump-probe, Acoustics multilayers, Acoustic resonators, Coherent phonons

## Abstract

Ultrahigh-frequency acoustic-phonon resonators usually require atomically flat interfaces to avoid phonon scattering and dephasing, leading to expensive fabrication processes, such as molecular beam epitaxy. Mesoporous thin films are based on inexpensive wet chemical fabrication techniques that lead to relatively flat interfaces regardless the presence of nanopores. Here, we report mesoporous titanium dioxide-based acoustic resonators with resonances up to 90 GHz, and quality factors from 3 to 7. Numerical simulations show a good agreement with the picosecond ultrasonics experiments. We also numerically study the effect of changes in the speed of sound on the performance of the resonator. This change could be induced by liquid infiltration into the mesopores. Our findings constitute the first step towards the engineering of building blocks based on mesoporous thin films for reconfigurable optoacoustic sensors.

## 1 Introduction

Coherent acoustic phonons, with frequencies in the GHz–THz range, have associated wavelengths between a few and hundreds of nanometers [1], [2], [3], [4], [5], [6], [7], [8], [9]. Among other applications, they are suitable for high resolution nanoimaging, non-destructive testing, and sensing. Acoustic phonon dynamics have been explored in systems such as plasmonic nanostructures, [10], [11], [12], [13] metasurfaces, [14] oxides, [15], [16], [17], [18] and semiconductor heterostructures [19], [20], [21], [22] with layer thicknesses on the nanometric scale [19], [23], [24]. To precisely tailor the nanophononic response and obtain high quality devices, expensive and complex growth and processing techniques are usually employed, including molecular beam epitaxy and electron beam lithography.

Conversely, mesoporous structures rely on cheap and reproducible bottom-up fabrication processes derived from soft chemistry, carried out in mild conditions, [25], [26] and are able to sustain gigahertz acoustic resonances [27], [28], [29], [30]. Brillouin light scattering experiments have been employed to demonstrate phononic band gaps in the 10–20 GHz range in periodic stacks of alternating layers of porous silicon dioxide (SiO_2_) and poly- (methyl methacrylate) (PMMA) [27], [31], [32]. More recently, mesoporous thin films (MTFs) based on SiO_2_ have been shown to support coherent acoustic modes between 5 and 100 GHz, with Q-factors ranging from 5 to 17. [29]. MTFs are also suitable for photonic sensing applications due to the high surface-to-volume ratio and tailorable mesopores [33], [34]. By liquid infiltration into the nanopores, a modulation of the optical and elastic properties of the material could be achieved, also enabling chemical functionalization in nanoacoustic devices [28], [35], [36].

A well-established material, with numerous applications for both the dense and mesoporous forms, is titanium dioxide (TiO_2_). Applications include photocatalysis, [37], [38] implants, [39] photovoltaics, [40] energy harvesting and storage, and sensing [41]. These materials are promising candidates for nanoacoustics. For instance, TiO_2_ anatase nanoparticles presented a particularly strong induced exciton shift when applying an acoustic strain pulse [42], [43]. Like SiO_2_/PMMA, phonon engineering has also been demonstrated with porous TiO_2_/PMMA multilayers [44].

In this work, we employ coherent phonon generation and detection techniques to study acoustic resonators based on TiO_2_ MTFs, with resonances up to 90 GHz. Our results indicate that GHz acoustic resonators based on mesoporous structures are not limited to SiO_2_, and open the possibility to more complex heterostructures formed by different materials. In addition, we theoretically investigate the effect of changes in the elastic properties of the mesoporous matrix on the acoustic resonances, which can be achieved via liquid infiltration into the pores. This platform constitutes a promising building block for developing environment-responsive nanosystems for nanoacoustic sensing and reconfigurable optoacoustic nanodevices based on soft and inexpensive fabrication methods.

## 2 Experiments and simulations

### 2.1 Sample fabrication

The TiO_2_ thin films are synthesized using the sol–gel method. The ordered mesoporosity is obtained using the evaporation-induced self-assembly of surfactants [25]. The fabrication details can be found in Ref. [29] for silica MTFs, in which the same approach is employed. Ethanolic solutions of TiCl_4_ are first prepared, and then the water is added; to obtain the porosity, the surfactant Pluronic F127 is finally added to the precursor solution. The final molar ratio of the solutions is TiCl_4_:H_2_O:EtOH 1:10:40 to obtain the dense films and TiCl_4_:F127:H_2_O:EtOH 1:0.005:10:40 to synthesize the mesoporous materials. These solutions are used immediately after preparation to deposit the films on top of clean Si substrates by dip coating.

**Fig. 1:**
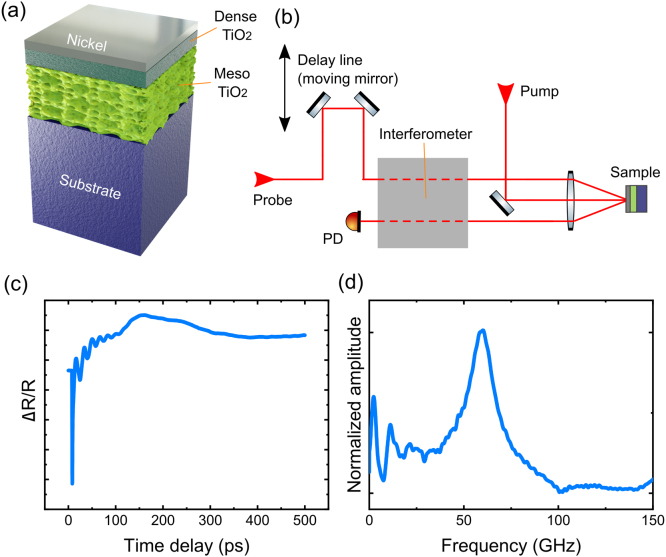
(a) Schematics of the mesoporous TiO_2_ sample design. (b) Experimental setup of the reflectometric pump-probe setup. An interferometric measurement can also be implemented, indicated by the gray box. [45], [46] (c) Transient reflectivity timetrace of the mesoporous TiO_2_ sample C. (d) Phononic spectrum corresponding to the timetrace shown in (c).

The schematics of the studied structure is shown in Fig. 1(a). First, a mesostructured titania film is deposited on the substrate and submitted to a stabilization process up to 200 °C to consolidate the oxide and stabilize the mesostructure [47]. Then, a capping layer of dense titania is synthesized and stabilized with the same treatment. Afterwards, the bilayer is submitted to a final calcination step of 2 h at 350 °C with a 1 °C/min heating ramp; this treatment is performed to eliminate the pores template and generate an accessible and interconnected mesoporosity [48]. Besides, it leads to a phase transition from amorphous titania to anatase [49]. After these steps, homogeneous and crack-free thin films of cm dimensions are achieved.

Afterwards, a ∼32 nm-thick nickel cover layer is deposited by vacuum thermal evaporation using a homemade system. The film thickness is measured during deposition by monitoring the frequency change of a resonant quartz crystal [29]. The dense layer is necessary to avoid nickel diffusion through the mesopores, and the Ni film acts as the acousto-optical transducer for coherent phonon generation and detection. [50]

Four samples are fabricated: three structures designed with different mesoporous layer thicknesses, defined by the dip-coating withdrawal speeds of 1, 2 and 3 mm/s, and one control sample without the mesoporous thin film. The dense layer withdrawal speed is set constant to 0.2 mm/s. The structures are respectively labeled as A, B, C, and Control. Table 1 presents the thickness and porosity parameters, derived from ellipsometry measurements (SOPRA GES5E ellipsometer). [51]

**Table 1:** Structural details of the layers from samples A, B, C and Control. Nominal (best fit) thickness values of the dense TiO_2_, nickel and mesoporous layers, and the porosity.

Sample	Mesoporous TiO_2_		Dense TiO_2_	Ni
	Thickness (nm)	Porosity (%)	Thickness (nm)	
A	103	48	14	32 (38)
B	144	40	26 (28)	32 (37)
C	191	44	25 (22)	32 (36)
Control	–	–	60	32 (34)

### 2.2 Coherent phonon generation and detection

The coherent acoustic phonon dynamics is studied via time-domain Brillouin scattering (TDBS) [52] in a pump-probe setup (see Fig. 1(b)). An incident pulsed pump laser (λ=758 nm, 200 fs pulse duration, 80 MHz repetition rate, 275 mW equivalent CW power), is focused with a 10-cm lens, and partially absorbed by the Ni transducer and excites acoustic phonons via photoinduced stress processes [52]. A second ultrafast laser pulse, namely the probe (same wavelength and pulse duration as for the pump pulse, and typically 5 mW power), delayed with respect to the pump, detects the instantaneous optical reflectivity modulated by the coherent acoustic phonons. Two different processes drive these modulations: the photoelastic interaction, i.e., the changes in the optical properties of the structure due to strain; and the surface displacement induced by the presence of phonons. For the first process, the coherent acoustic phonons induce a change in the index of refraction in the structure proportional to the strain, which will then modulate the reflectivity of the probe. For the latter process, surface displacement detection is performed by implementing a Sagnac interferometer [45], [46]. Further experimental details can be found in Ref. [29]. The reflectivity setup is simpler in terms of optical alignment. However, depending on the studied materials and layer ordering, the modulation of the photoelastic properties is weak and the detection of coherent acoustic phonons becomes impossible. In such cases, the interferometric setup must be employed. [45]

Fig. 1(c) depicts a typical transient reflectivity timetrace in the interferometric configuration for a TiO_2_-based mesoporous sample. Coherent oscillations are visible for time delay < 120 ps. They result from longitudinal coherent phonons modulating the optical properties of the structure. By performing a Fourier transform, we extract the phononic response in the frequency domain (Fig. 1(d)).

### 2.3 Numerical simulation

To investigate the acoustic resonances, we simulate the acoustic strain, displacement, and electric fields by implementing a transfer matrix method to solve the respective wave equations for a multilayered structure with contrasting refractive indices and acoustic impedances [53], [54], [55], [56], [57]. The solutions for the wave equation take into account the boundary conditions related to the continuity of the atomic displacement ($u\left(z\right)$) and stress ($\frac{\partial u\left(z\right)}{\partial z}$) at the interfaces, as well as a zero-strain condition at the cap layer-air interface [57]. By calculating the normalized optical and acoustic solutions we can simulate the phonon generation spectrum by integrating the strain, the electric field modulus square and the photoelastic constant. The phonon detection is then obtained by an overlap integral of the generation spectrum with the photoelastic constant, the strain, and the electric field square [54], [55], [56]. The material parameters used in the simulations are shown in Table 2. The surface displacement is also simulated by taking the product of the solutions of the phonon displacement amplitude at the interface between the air and the last layer of the structure, and the phonon generation spectrum. It is assumed that the optical absorption and coherent phonon generation are entirely limited to the nickel transducer layer.

**Table 2:** Optical and elastic properties of the studied materials for the numerical simulation. For the mesoporous TiO_2_, the index of refraction is derived from the Bruggemann approximation for a mixture of two dielectric media states [58]: dense TiO_2_ matrix and the air (pores), according to the porosity on Table 1.

Material	Index of refraction	Speed of sound (m/s)	Density (g/cm^3^)
TiO_2_	2.56	6700	2.9
SiO_2_	1.5375	5750	2.2
Air	1.00028	343	1.275E−3
Nickel	2.218+4.893i	5580	8.908
TiO_2_ (mesoporous)	(1.76 – 1.90) [58]	3886, 3363, 4221^a^	1.51, 1.74, 1.62^a^
Si	3.72	8433	2.32

a For samples A, B and C, respectively. Refer to main text for more details.

## 3 Results and discussion

### 3.1 Surface displacement and photoelastic interaction

**Fig. 2:**
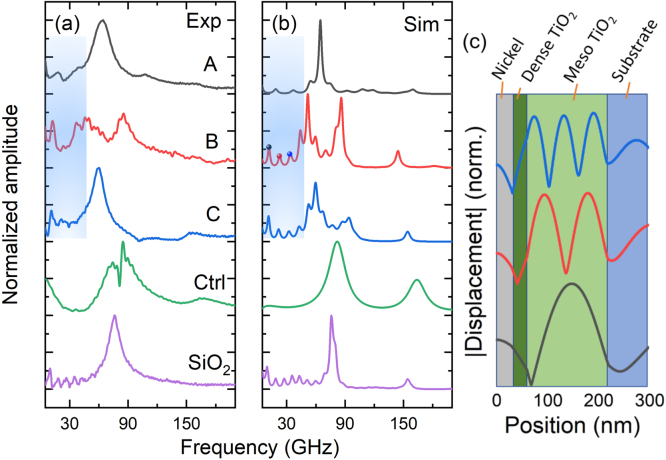
(a) Experimental and (b) simulated spectra of the surface displacement of TiO_2_-based samples A, B, C and Control, and SiO_2_-based sample C (Ref. [29]), for comparison. The low-frequency-shaded areas (up to 50 GHz) indicate the first confined modes in the mesoporous layer. (c) Displacement field of the modes at 13, 25.5 and 38 GHz on TiO_2_ sample B, respectively indicated as black, red and blue circles in (b). (For interpretation of the references to color in this figure legend, the reader is referred to the web version of this article.)

The experimental surface displacement spectra of the TiO_2_-based mesoporous samples A, B and C, and the control sample without mesoporous layer, are presented in Fig. 2(a). An intense and broad peak is present between 55 GHz and 90 GHz, and a weaker peak around 150–180 GHz is also resolved. These features are associated to the fundamental mode and the first harmonic of an acoustic resonance in the nickel and dense TiO_2_ bilayer [29]. At lower frequencies, low amplitude peaks are resolved, which correspond to coherent acoustic phonons confined in the mesoporous layer. Samples A, B, and C have fundamental modes at 17.24 ± 0.21 GHz, 12.63 ± 0.02 GHz, and 11.54 ± 0.04 GHz, respectively. In a Fabry–Perot mode, the resonance frequency should be proportional to the inverse of the spacer thickness. The observed resonances follow this inverse order of the mesoporous thickness (refer to Table 1), indicating phonon confinement. The results for the mesoporous SiO_2_ sample, reported in Ref. [29], are also displayed in Fig. 2(a,b), for comparison, where the acoustic modes at the mesoporous layer are better resolved. In contrast, the Control structure, without a mesoporous layer, does not show any resonances below 40 GHz, as expected. The spectrum associated to this sample presents a peak at 84.8 GHz, corresponding to the resonance on metallic and dense layers, with a dip at 81.2 GHz, probably originating from a destructive interference between the surface displacement and Brillouin scattering contributions of the signal.

The simulation of the surface displacements are shown in Fig. 2(b). A good agreement between the experimental data and simulation results is achieved. The interference dip present in the Control sample is not reproduced in the simulation as Brillouin scattering is not considered simultaneously with the surface displacement effect. Furthermore, the experimental results of sample C show a dip at ∼ 100 GHz.

**Fig. 3:**
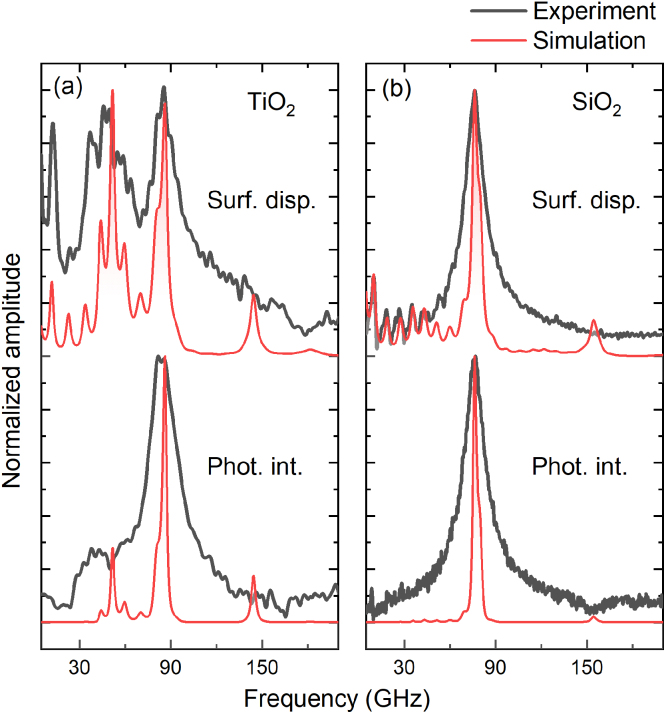
Experimental results (black line) and the respective transfer matrix method (TMM) simulations (red area) on surface displacement and photoelastic interaction for (a) TiO_2_ sample B and (b) SiO_2_ sample C, from Ref. [29]. Acoustic modes at the low-frequency region are only present in the surface displacement spectra for both TiO_2_ and SiO_2_. (For interpretation of the references to color in this figure legend, the reader is referred to the web version of this article.)

In order to get a more detailed picture of the phononic behavior of these structures, it is worth comparing the signals -measured and calculated- corresponding to the surface displacement (interferometry) and modulation of the index of refraction (reflectometry, photoelastic effect). Figs. 3(a) and (b) display the phononic spectra of both modulation effects for the TiO_2_ (sample B) and SiO_2_ structures, respectively. For both materials, the intense peak is present in the two cases. However, the confined modes at lower frequency are observed uniquely in the interferometric dataset, in which the first five acoustic resonances at 12.6, 23.9, 36.9, 47.7 and 57.9 GHz have respective quality factors of 2.7, 4.7, 4.1, 3.9 and 6.9, within a 10% error.

Simulations for the surface displacement and the photoelastic interaction for both structures are displayed as red lines in Figs. 3(a) and (b). They reproduce the main peaks of the experimental datasets for both cases. Furthermore, the low-frequency modes in the photoelastic interaction calculation, associated to the mesoporous resonances, are negligible when compared to the interferometric simulation, in accordance with the experimental results. The higher frequency peaks are considerably narrower in the simulation, compared to the experimental results. The broadening of the modes is mainly related to leakage into the substrate, surface roughness and acoustic losses. The latter two are not considered in the simulation.

The lack of a photoelastic interaction contribution in both experiment and simulation supports the hypothesis that the respective vibrational resonances are confined in the mesoporous layer. Note that the mesoporous material is transparent for the incident laser wavelength -with an associated weak photoelastic constant-. For the surface displacement results, the acoustic modes in the soft mesoporous layer leads to vibrations within the whole structure, thus, contributing to the interferometric detection.

### 3.2 Surface displacement dependence on TiO_2_ speed of sound

Elastic properties of TiO_2_ thin films, such as mass density (ρ), Young’s modulus (E) and speed of sound (v) depend on several factors such as fabrication method, layer thickness, annealing temperature and crystallinity. This dependence leads to a broad range of reported values for ρ (from 2.9 to 3.9 g/cm^3^) and E (from 80 to 250 GPa), for TiO_2_ in its anatase phase [59], [60], [61], [62], [63], [64], [65], [66]. The speed of propagation of longitudinal acoustic modes is calculated according to:

(1) $$v=\sqrt{\frac{E\left(1-\nu \right)}{\rho \left(1+\nu \right)\left(1-2\nu \right)}},$$

where ν is the Poisson ratio. Considering a Poisson ratio of 0.27 for TiO_2_, the broad range of values for E and ρ leads to inaccurate values of longitudinal acoustic wave speed, spanning from 5500 to 11,000 m/s. A precise determination of these parameters is out of the scope of this work, however a quantitative analysis is required for the numerical simulations and further discussion.

**Fig. 4:**
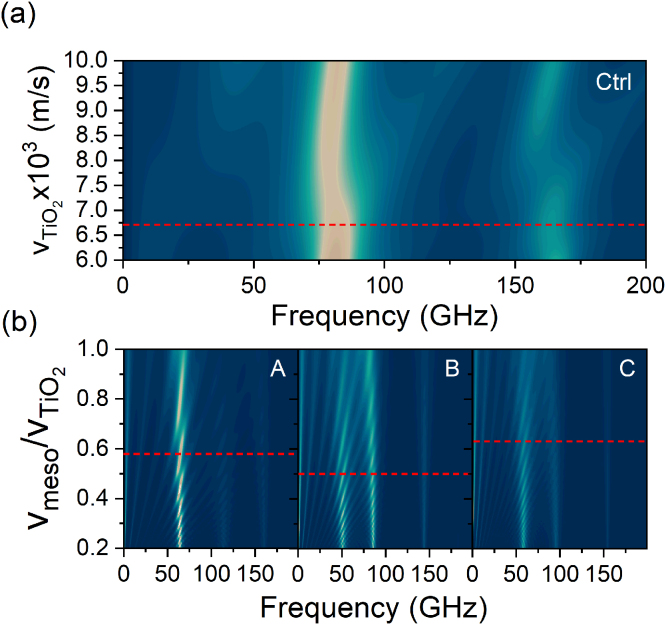
Colorplot of surface displacement amplitude vs frequency and (a) dense TiO_2_ speed of sound for control sample, and (b) speed of sound ratio between mesoporous and dense materials for mesoporous TiO_2_-based samples. The dashed lines at V${}_{\mathrm{TiO}2}=6700\;\mathrm{m/s}$ and V${}_{meso}/$V${}_{\mathrm{TiO}2}$ = 0.58, 0.50 and 0.63, for samples A, B and C, respectively, correspond to the values that best match with experimental results.

We simulate the surface displacement at different TiO_2_ sound speeds for the control sample, within the range of 6000 to 10,000 m/s, and present the results on Fig. 4(a), in a colorplot. The spectrum that best matches with the experiment is simulated with v${}_{{\mathrm{TiO}}_{2}}=6700$ m/s, indicated by a dashed red line. The density used in the simulation is 2.9 g/cm^3^.

The mesoporous TiO_2_ layer parameters are extracted according to the layer porosity and the dense TiO_2_ constants. The refractive index is calculated using the Bruggemann approximation of a complex composite by an effective homogeneous medium for a mixture of two dielectric media [58]. The mass density is obtained from a direct relation between the bulk TiO_2_ density and the material porosity (see Table 1), and the value for each sample is displayed in Table 2. The speed of sound in the mesoporous material is adjusted to match the experimental results for samples A and C. For sample B we fitted Lorentzian peaks from the surface displacement data to extract the frequency of the mesoporous first five harmonics and calculated the sound speed (v=2hf) considering the fundamental frequency f, obtained from the linear regression fitting, and the mesoporous layer thickness h=144nm. The thickness of the dense oxide and the nickel layers are modified so that the simulated high-frequency peak match with the experiment. We also consider acoustic losses with an effective phonon decay length of ∼75nm for the MTFs. [29], [67]. The evolution of the surface displacement spectrum as a function of the ratio between the sound velocities of the mesoporous and dense TiO_2_ layers for the three samples is shown on the three panels of Fig. 4(b). The dashed red lines indicate the ratio of the sound velocities for which the simulations best match with the experimental results, and the respective spectra are displayed in Fig. 2(b). They exhibit a good agreement with the experiment. Considering the mesoporous speed of sound obtained from this analysis (see Table 2), the density, and the Poisson ratio for mesoporous materials ν=0.2, we obtain the following Young’s modulus of the thin films: ∼20.52GPa, ∼17.71GPa, and ∼25.97GPa, for samples A, B and C, respectively. Note that these values differ from those reported in the literature for titania mesoporous thin films, between 35 and 50 GPa, [68], [69] obtained with similar fabrication methods but measured with different techniques.

In Fig. 4(b) we observe a clear increase in mode frequency for the low-frequency modes upon raising the speed of sound in the mesoporous layer. In contrast, the high-frequency modes are hardly affected. This implies that the low-frequency resonances are indeed mainly confined within the MTF. It is worth mentioning that the studied variation of the sound velocity in the mesoporous layer can be experimentally achieved by liquid infiltration into the nanopores, which modifies the elastic properties of the effective medium composed of the TiO_2_ dense matrix and air in the pores. As chemical adsorption and capillary condensation are reversible processes, MTFs can be employed as active elements in optoacoustic sensing devices.

The results exhibited in this section are compatible with what has been reported on SiO_2_ mesoporous systems, [29] and reinforce the feasibility of coherent acoustic phonon generation and detection in different mesoporous materials. Furthermore, this work reinforces the concept of mesoporous thin films as potential environment-responsive platforms able to transduce a physicochemical process (adsorption, capillary condensation) into optical [26], [33], [34] or nanoacoustic signals [29]. This building block represents an important step towards the engineering of more complex structures based on such soft materials for practical applications, e.g., sensing.

## 4 Conclusion

In this work we have demonstrated the confinement of gigahertz coherent acoustic phonons in mesoporous titanium dioxide structures, extending the concept of mesoporous acoustic resonators to different material systems. Simulations on photoelastic interaction and surface displacement convey appreciable agreement with the experimental results. Furthermore, we discussed the effects of changes in the elastic parameters on the mesoporous resonator performance. These changes can be induced by chemical compound infiltration. This concept can be extended and applied to novel sensing devices based on ultrahigh-frequency resonators, with the mesoporous layer as the active optoacoustic element. Our findings unlock the way to a promising platform for nanoacoustic sensing and reconfigurable optoacoustic nanodevices based on soft, inexpensive fabrication methods.

## CRediT authorship contribution statement

**E.R. Cardozo de Oliveira:** Performed the simulations, Data curation. **C. Xiang:** Performed the simulations, Data curation. **M. Esmann:** Performed the simulations, Data curation. **N. Lopez Abdala:** Fabricated and characterized the samples, Performed the simulations, Data curation. **M.C. Fuertes:** Fabricated and characterized the samples. **A. Bruchhausen:** Fabricated and characterized the samples. **H. Pastoriza:** Fabricated and characterized the samples. **B. Perrin:** Performed the picosecond ultrasonics experiments. **G.J.A.A. Soler-Illia:** Conceptualization, Directed the research, Fabricated and characterized the samples. **N.D. Lanzillotti-Kimura:** Conceptualization, Directed the research, Performed the picosecond ultrasonics experiments, Performed the simulations, Data curation.

## Declaration of Competing Interest

The authors declare that they have no known competing financial interests or personal relationships that could have appeared to influence the work reported in this paper.

## Data Availability

Data will be made available on request.

## Appendix A Supplementary data

MMC S1 Experimental results and transfer matrix simulations of photoelastic interaction and surface displacement for samples A and C. We highlight that the low-frequency modes, associated to the phonon confinement in the mesoporous layer, are not apparent in the photoelastic detection, whereas they are well resolved for the surface displacement.
