# Supplementary material for: Probing gigahertz coherent acoustic phonons in TiO2 mesoporous thin films
Source: Photoacoustics. 2023 Mar 4;30:100472. doi: 10.1016/j.pacs.2023.100472 (PMC10026033; doi:10.1016/j.pacs.2023.100472)
Supplement: MMC S1 — Experimental results and transfer matrix simulations of photoelastic interaction and surface displacement for samples A and C. We highlight that the low-frequency modes, associated to the phonon confinement in the mesoporous layer, are not apparent in the photoelastic detection, whereas they are well resolved for the surface displacement. [file mmc1.pdf]

Supplementary Information

Probing Gigahertz Coherent Acoustic Phonons in TiO_2 Mesoporous Thin Films

E. R. Cardozo de Oliveira[a], C. Xiang[a], M. Esmann[a,b], N. Lopez Abdala[c], M. C. Fuertes[d], A. Bruchhausen[e], H. Pastoriza[e], B. Perrin[f], G. J. A. A. Soler-Illia[c], N. D. Lanzillotti-Kimura[a]

[a]Université Paris-Saclay, CNRS, Centre de Nanosciences et de Nanotechnologies, 91120 Palaiseau, France

[b]Institute for Physics, University of Oldenburg, 26129 Oldenburg, Germany

[c]Instituto de Nanosistemas, Escuela de Bio y Nanotecnologías, Universidad Nacional de San Martín-CONICET, Buenos Aires, Argentina

[d]Gerencia Química, Inst. de Nanociencia y Nanotecnología, CNEA-CONICET, Buenos Aires, Argentina

[e]Centro Atómico Bariloche, Inst. de Nanociencia y Nanotecnología, CNEA-CONICET, Rio Negro, Argentina

[f]Sorbonne Université, CNRS, Institut des NanoSciences de Paris, INSP, F-75005 Paris, France

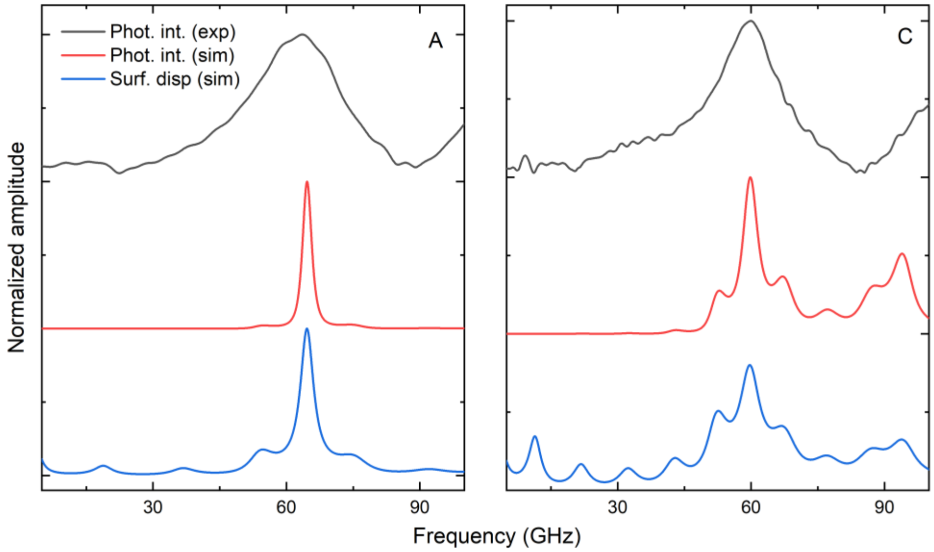

Fig S1: Experimental results (black) and respective TMM simulations of normalized photoelastic interaction (red) and surface displacement (blue) for samples A and C.

Figure S1 shows the experimental results (black) and TMM simulations of normalized photoelastic interaction (red) and surface displacement (blue) for samples A and C. Note that the low-frequency modes, associated to the phonon confinement in the mesoporous layer, are not apparent in the photoelastic detection, whereas they are well resolved for the surface displacement.
